# Manuka honey and its component, methyl syringate, shift neutrophil release profiles from pro-inflammatory while preserving pro-regenerative growth factor release *in vitro*

**DOI:** 10.3389/fimmu.2026.1760968

**Published:** 2026-03-03

**Authors:** Evan N. Main, Samantha C. Hall, Gary L. Bowlin

**Affiliations:** Department of Biomedical Engineering, University of Memphis, Memphis, TN, United States

**Keywords:** biomaterial additives, host-biomaterial response, immunomodulation, neutrophil, tissue regeneration

## Abstract

**Introduction:**

Neutrophils, traditionally viewed as short-lived effector cells of acute inflammation, are now recognized as multifunctional contributors to immune regulation, tissue repair, and pathology. Upon activation, they elicit robust oxidative and cytokine responses, including the release of myeloperoxidase (MPO) and interleukin-8 (IL-8), which amplify neutrophil recruitment, prolong survival, and reinforce inflammatory signaling. Neutrophils also secrete regenerative mediators, including hepatocyte growth factor (HGF), vascular endothelial growth factor A (VEGF-A), and matrix metalloproteinase-9 (MMP-9). Manuka honey and its principal phenolic constituent, methyl syringate, have recently been shown to reduce neutrophil inflammatory activity, including intracellular reactive oxygen species (ROS) production and neutrophil extracellular trap formation (NETosis). However, their effects on primary human neutrophil signaling, enzyme release, and growth-factor secretion have not been characterized.

**Methods:**

To address this, peripheral blood neutrophils were isolated from healthy donors using density gradient separation and seeded into 96-well plates. Cells were stimulated with PMA and treated for 3 or 6 hours with 5% or 10% Manuka honey or 600 or 1300 µM methyl syringate; unstimulated cells served as negative controls, and PMA-stimulated cells served as positive controls. Supernatants were collected and analyzed using magnetic bead-based multiplex ELISAs.

**Results:**

Both Manuka honey and methyl syringate reduced the release of inflammatory mediators in PMA-activated neutrophils, with dose- and time-dependent effects. Most treatments significantly reduced MPO levels at 3 hours and, across all treatments, at 6 hours, typically achieving ≥50% reductions and ≥70% suppression at higher doses. IL-8 release showed the most potent and most consistent inhibition, with Manuka honey reducing levels to near baseline by 6 hours. MMP-9 showed modest responsiveness, particularly to methyl syringate. HGF secretion remained unchanged across treatments. VEGF-A release was markedly decreased by Manuka honey at both time points (≥70%), whereas methyl syringate produced more minor but statistically significant reductions only at 6 hours.

**Discussion:**

In conclusion, the data suggest that Manuka honey and methyl syringate are both efficacious at reducing pro-inflammatory cytokines and enzymes. However, methyl syringate alone preserved factors associated with pro-angiogenic and remodeling despite reduced inflammation.

## 1 Introduction

Biomaterials designed for tissue engineering and regenerative medicine aim to restore or replace damaged tissue while integrating seamlessly with host biology. Their success depends not only on structural and mechanical performance but also on their ability to modulate the host immune response. Regardless of design sophistication, no biomaterial is truly “inert”; all implanted materials are perceived as foreign by the innate immune system. The early immune response, directed both at the implant and at the injury created during implantation, plays a decisive role in determining whether the outcome is constructive tissue regeneration or continuous inflammation and eventually fibrosis (1).

Among the first cells to respond to biomaterial implantation are neutrophils, which rapidly migrate to the injury site upon sensing damage-associated molecular patterns (DAMPs) and plasma proteins adsorbed to the biomaterial surface (2). Once regarded as short-lived phagocytes with limited regulatory roles, neutrophils are now recognized as key modulators of inflammation and tissue regeneration. They influence macrophage polarization, fibroblast recruitment, angiogenesis, and extracellular matrix remodeling (3). A balanced neutrophil response is essential for clearing debris and initiating repair; however, sustained or excessive activation can impair resolution, hinder integration, and initiate fibrotic encapsulation (4).

Neutrophil activity in the biomaterial microenvironment can be broadly categorized into several interconnected functions. First, neutrophils can undergo NETosis, a specialized form of cell death in which chromatin decondenses and is expelled as neutrophil extracellular traps (NETs) containing DNA, histones, and granule enzymes such as neutrophil elastase (NE) and myeloperoxidase (MPO). Excessive NET deposition can coat implant surfaces, impede cellular infiltration, and prolong inflammation (3, 5–7). Second, neutrophils produce large amounts of ROS via NADPH oxidase and MPO, which serve antimicrobial roles but can also oxidatively modify adsorbed proteins, further induce pro-inflammatory redox signaling, and damage the biomaterial interface (8–11). Lastly, activated neutrophils release a suite of cytokines and chemokines, including interleukin (IL)-1β, IL-6, TNF-α, and CXCL8 (IL-8), that amplify leukocyte recruitment and reinforce pro-inflammatory signaling.

Intracellular ROS act as signaling intermediates that activate the inhibitory κB kinase (IKK) and the nuclear factor κB (NF-κB) pathway, leading to transcription of pro-inflammatory cytokines and perpetuating leukocyte recruitment (12). Antioxidant therapeutics can attenuate this cascade, reducing NF-κB activation and downstream inflammatory gene expression (13). ROS generation is also an essential precursor for NETosis, promoting nuclear translocation of NE and MPO to mediate chromatin decondensation (14). Through these oxidative, proteolytic, and signaling pathways, neutrophils play a central role in determining whether a wound or a biomaterial’s microenvironment progresses toward regeneration or prolonged inflammation.

In parallel with advances in biomaterial design, researchers have renewed interest in natural bioactive compounds that can modulate excessive inflammation. Among these, Manuka honey, derived from *Leptospermum scoparium*, has emerged as a promising adjunctive therapeutic. Over the past three decades, Manuka honey has demonstrated robust antibacterial, anti-inflammatory, and antioxidant properties relevant to wound-healing and tissue-engineering applications (15, 16). Its potent antibacterial activity is attributed primarily to methylglyoxal (MGO), a reactive dicarbonyl compound that is effective against antibiotic-resistant organisms (17). Additionally, the honey’s high sugar concentration generates a strong osmotic gradient, promoting autolytic debridement and maintaining a moist wound environment conducive to regeneration (17).

**Figure 1:**
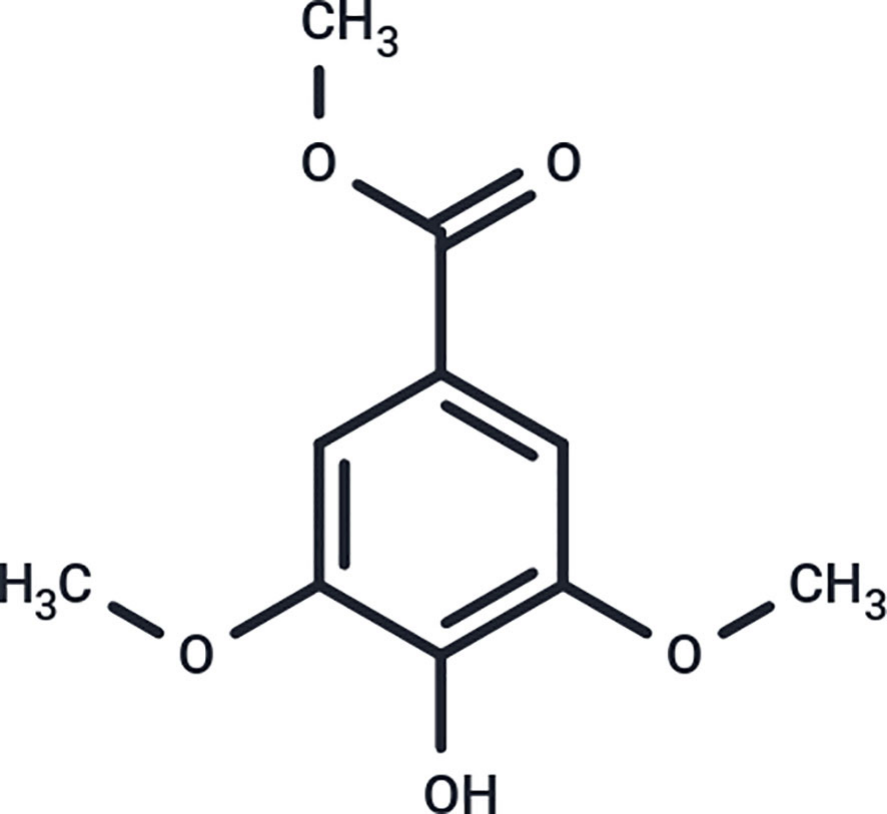
Chemical structure of methyl syringate.

Beyond antimicrobial effects, Manuka honey exhibits significant immunomodulatory properties. It reduces neutrophil activation and inflammatory signaling within a defined therapeutic range and retains these properties when incorporated into fibrous electrospun biomaterials (18, 19). In differentiated neutrophil-like dHL-60 cells, Manuka honey has been shown to suppress NF-κB activation, inhibit chemotaxis, and decrease secretion of certain pro-inflammatory cytokines (20, 21). The anti-inflammatory and antioxidant effects of Manuka honey have recently been shown to arise from its abundant phenolic constituent, methyl syringate (**Figure 1**). Methyl syringate has demonstrated potent inhibition of neutrophil NETosis and intracellular ROS activity, in some cases exceeding the corresponding concentrations of whole Manuka honey (16). In doing so, Manuka honey and its constituent phenolic compound, methyl syringate, can directly modulate key neutrophil effector functions, mitigating oxidative and proteolytic damage at the biomaterial interface and NET-associated signaling.

Despite these promising findings, a critical piece of the puzzle remains to be elucidated. As previously mentioned, neutrophils are not solely effector cells, but drivers and coordinators of the acute inflammatory response to injury and biomaterial implantation. While Manuka honey and methyl syringate have been shown to reduce immediate effector responses in neutrophils, their effects on upstream inflammatory signaling release profiles remain incompletely explored.

Thus, the aim of this study was to identify key signaling components released by pro-inflammatory-primed neutrophils and to determine the effects of whole Manuka honey and isolated methyl syringate at varying concentrations, based on their success in previous studies in reducing inflammatory effector functions, on the overall *in vitro* release profile *(*16*).* While the full extent of the neutrophil secretome remains to be elucidated, this investigation focuses on select proteins, enzymes, and growth factors associated with neutrophil-mediated inflammation, as well as newly identified roles of neutrophils in pro-regenerative processes necessary for proper healing. Below, several of these objective proteins and their importance in wound healing and biomaterial integration are discussed.

Myeloperoxidase (MPO) is a heme-containing enzyme abundantly expressed in neutrophil azurophilic granules, where it plays a central role in antimicrobial defense and inflammation. During activation, MPO catalyzes the conversion of hydrogen peroxide and chloride ions into hypochlorous acid (HOCl), a potent oxidant that contributes to pathogen clearance within the innate immune response (22, 23). While essential for microbial killing, sustained MPO activity can amplify oxidative stress, prolong inflammation, and promote tissue injury, complicating resolution processes (24).

MPO also regulates key aspects of neutrophil biology. It delays neutrophil apoptosis, extending their lifespan at inflammatory sites and driving tissue damage by perpetuating acute inflammation (25, 26). At the cellular interface, MPO modulates β_2_ integrins, such as CD11b/CD18, facilitating neutrophil adhesion and transmigration across the endothelium during vascular inflammation, a process implicated in numerous pathologies, including pulmonary edema and vascular graft implant failure (27–29). Elevated MPO release is associated with a range of pathologies, including cardiovascular and autoimmune diseases (30, 31). In acute coronary syndrome, plasma MPO levels correlate with the extent of vascular inflammation and leukocyte activation, supporting its value as a biomarker for systemic inflammatory burden (32). Beyond its inflammatory functions, MPO can influence tissue repair dynamics. Although early MPO activity aids in pathogen clearance, excessive or deregulated production can impair epithelial proliferation and delay wound closure (33). Balancing MPO activity with pro-resolving mediators is therefore critical for transitioning from a dysregulated acute inflammatory response toward regeneration (34, 35). Collectively, MPO plays a dual role, both a defender against infection and a potential driver of chronic inflammation. Therapeutic strategies that fine-tune MPO activity may help mitigate oxidative tissue damage while preserving essential antimicrobial functions.

Matrix metalloproteinase-9 (MMP-9) is a neutrophil-derived enzyme that plays a central role in the progression of inflammation and tissue remodeling. Released from tertiary granules during activation, MMP-9 degrades extracellular matrix components, enabling neutrophils to traverse vascular and interstitial barriers and rapidly infiltrate injured tissue (36). By interacting with β_2_ integrins such as CD18, MMP-9 further enhances chemotaxis and directional migration in response to inflammatory cues (37, 38). MMP-9 also contributes to the amplification of inflammation. Upon release, it can stimulate surrounding stromal and immune cells to produce additional cytokines and chemokines, reinforcing leukocyte recruitment and intensifying inflammatory signaling (39). Elevated MMP-9 levels correlate with disease severity in several inflammatory pathologies, including glomerulonephritis, where enhanced neutrophil infiltration parallels increased enzymatic activity (40).

However, MMP-9 activity is not exclusively detrimental. While excessive matrix degradation drives tissue injury, chronic inflammation, and fibrosis, as seen in conditions like cystic fibrosis and rheumatoid arthritis, controlled MMP-9 activity supports the transition to tissue repair (41, 42). During the resolution phase, MMP-9 facilitates extracellular matrix remodeling, angiogenesis, and restoration of tissue architecture, aligning neutrophil activity with regenerative processes (43). MMP-9 and ECM interactions are also significant, as they mediate the release of matrix-bound factors, notably vascular endothelial growth factor (VEGF), critical for angiogenesis and tissue repair (44). Therefore, MMP-9 not only contributes to ECM degradation, but it also plays a key role in the availability of growth factors that support cellular migration and differentiation (45). MMP-9 functions as a dual-edged sword in neutrophil-driven inflammation. While it accelerates recruitment and amplifies inflammatory signaling, it also supports matrix remodeling during healing. Its impact is therefore context-dependent, and therapeutic strategies that modulate MMP-9 activity may help limit tissue damage while preserving its beneficial roles in repair.

Interleukin-8 (IL-8), a central CXC chemokine, mediates neutrophil activation and recruitment during inflammation. Produced by monocytes, epithelial cells, endothelial cells, and neutrophils themselves in response to stimuli, IL-8 acts through CXCR1 and CXCR2 to guide neutrophil migration to sites of infection or tissue injury (46, 47). Elevated IL-8 levels are characteristic of numerous inflammatory diseases, including severe COVID-19, in which IL-8 promotes a prothrombotic neutrophil phenotype (48). Physiologically, stimulation by IL-8 or, *in vitro*, by phorbol myristate acetate (PMA) can induce neutrophils to release more IL-8, generating a self-amplifying, positive-feedback loop that sustains inflammation (49). IL-8 signaling involves phosphorylation of ERK and PI3K pathways, coordinating directed chemotaxis and promoting neutrophil survival (50, 51). Collectively, IL-8 serves as both a key mediator and biomarker of inflammation and represents a promising therapeutic target for controlling excessive neutrophil-driven tissue damage in a variety of pathologies (49, 52).

Recent evidence suggests that neutrophil extracellular traps (NETs), traditionally associated with antimicrobial defense, may also participate in tissue regeneration. NET components can modulate local inflammation and facilitate transitions to pro-reparative immune phases (53). Furthermore, neutrophil-mediated clearance of apoptotic cells prevents sustained inflammatory signaling and promotes an environment conducive to proper healing (54). Thus, modulating neutrophil activity (but not completely negating it) is a promising therapeutic strategy to enhance regenerative outcomes. Maintaining this balance between inflammatory and reparative phenotypes is crucial as prolonged inflammation disrupts tissue repair, whereas its complete absence prevents healing.

Neutrophils are now becoming recognized for their pro-regenerative roles in tissue repair following injury. This evolving perspective highlights their functional diversity and the innate immune system’s capacity to support regeneration rather than solely mediate inflammation. A key mechanism underlying neutrophil-driven regeneration is their ability to adopt a pro-reparative phenotype that influences the immune microenvironment. Neutrophils have been shown to induce macrophage polarization toward a pro-regenerative state, which is essential for effective tissue healing. In hepatic injury models, neutrophil depletion results in a marked reduction in reparative macrophages and impaired regeneration after tissue damage (55). Neutrophils also release growth factors and cytokines that aid tissue growth and regeneration (53).

Thus, this study sought to determine whether methyl syringate and Manuka honey’s ability to reduce neutrophil-mediated inflammation would affect their capacity to release anti-inflammatory cytokines and pro-regenerative growth factors that are critical to proper wound healing (56). Previous literature has made it clear that excessive neutrophil inflammation is deleterious to wound healing and biomaterial integration; however, neutrophil-deficient or knockout models fail to achieve adequate wound healing (57, 58). Therefore, neutrophil involvement in the host-biomaterial niche should not be avoided entirely, either through knockout or through neutrophil apoptosis induction, but a balance must be struck to avoid dysregulation on either side.

Neutrophils are critical immune effector cells that contribute to the release of hepatocyte growth factor (HGF), a mediator essential for tissue repair and regeneration in organs such as the liver and lungs. Multiple studies have characterized the mechanisms governing neutrophil-derived HGF and its biological impact. HGF drives mitogenic, morphogenic, and angiogenic responses in epithelial and endothelial cells, thereby facilitating revascularization at the injury site (59). Following tissue injury, including hepatic damage, neutrophil recruitment is promoted by chemokines such as CXCL1, produced by hepatocytes in a STAT3-dependent manner. Recruited neutrophils subsequently release HGF, supporting hepatocyte proliferation and accelerating liver regeneration (60). Neutrophils also serve as an essential source of HGF in the lung. During acute respiratory failure, both circulating and alveolar neutrophils produce HGF, contributing to local tissue repair by modulating inflammatory responses (61). For example, efferocytosis of apoptotic cell debris and extracellular vesicles by neutrophils in a post-partial hepatectomy model induced an activated phenotype. Still, classical inflammatory responses such as NETosis, ROS respiratory burst, degranulation, or secretion of pro-inflammatory cytokines were not upregulated. Instead, neutrophils released various growth factors, including HGF, which contributed to functional tissue regeneration (62).

In addition to HGF release, neutrophils are key contributors to VEGF-A release both directly and through ECM-MMP-9 interaction, a central mediator of angiogenesis and inflammation. As significant sources of VEGF-A, neutrophils support endothelial cell proliferation and migration during neovascularization. Storage of VEGF-A within neutrophil intracellular granules enables rapid release upon activation. This pool can be mobilized by stimuli such as PMA, thereby promoting endothelial permeability and tubule formation during angiogenic responses (63–65). Neutrophil-derived VEGF-A also contributes to immune regulation. VEGF-A enhances neutrophil migration and activation, and signaling through VEGFR1 on neutrophils can trigger additional VEGF release, thereby amplifying inflammatory responses through a positive feedback loop (66). VEGF-A, in a complex interplay with MMP-9, can promote remodeling and vascularization of damaged tissues by MMP-9 clearing pathways in the extracellular matrix for VEGF-A to then develop blood vessels along these pathways (67). However, a prolonged and dysregulated release of MMP-9 and VEGF-A is also associated with pathological inflammation (68).

The interdependence among the various neutrophil release factors, both inflammatory and regenerative, further highlights the importance of balance in the acute phase of inflammation and indicates that it is far more nuanced than simply maximizing or minimizing overall neutrophil behavior. Therefore, the hypotheses of this study were two-fold. Given the efficacy of Manuka honey in both wound-healing and anti-inflammatory applications, Manuka honey and methyl syringate would inhibit the signaling mechanisms by which neutrophils perpetuate a pro-inflammatory cascade that prolongs acute-phase inflammation to the point of pathology. Second, if these compounds reduce neutrophil inflammatory signals, the pro-regenerative functions neutrophils perform could be preserved. As such, two of the best-performing concentrations of both methyl syringate and Manuka honey in terms of effector function (intracellular ROS and NETosis) mediation were selected for this study, based on previous investigations. Additionally, based on oxidation levels compared to untreated controls and viability assays, these concentrations did not adversely affect cellular viability in either HL60-derived cell line or primary neutrophils (16).

## 2 Materials and methods

### 2.1 Neutrophil isolation

Five independent experiments were conducted using freshly isolated peripheral blood neutrophils obtained from five healthy adult donors (3 female, 2 male) of randomized race and sex. Exclusion criteria included autoimmune, endocrine, cardiovascular, or inflammatory disease, and tobacco use. Donors abstained from alcohol and non-steroidal anti-inflammatory drugs (NSAIDs) for 48 h before donation and fasted for ≥12 hours (69–75). Donor recruitment, phlebotomy, experimental procedures, and data handling were performed in accordance with University of Memphis Institutional Review Board approval (IRB ID: PRO-FY2020-230), including written informed consent (16).

Neutrophils were isolated using a validated density-gradient separation protocol that reliably yields ≥96% pure neutrophils (16, 76, 77). Whole blood was collected into EDTA vacutainers (BD, Franklin Lakes, NJ, USA; #366643), and autologous serum was collected into untreated serum tubes (BD; #366668). Following gravitational separation into leukocyte and erythrocyte fractions, the leukocyte layer was aspirated and centrifuged at 200 × g for 10 min at ambient temperature (Sorvall ST8, Rotor 75005701; Thermo Scientific). The supernatant was discarded, and the pellet was resuspended in PBS and layered over 3 mL Isolymph (CTL, Deer Park, NY, USA; density 1.077 ± 0.001 g/mL; #759050), followed by centrifugation at 300 × g for 40 min at ambient temperature with brake disabled. Monocytes were removed, and the remaining fraction was subjected to hypotonic lysis by resuspending in ice-cold 0.2% NaCl for 30 s, followed by restoration of isotonicity using ice-cold 1.6% NaCl. NaCl solutions were prepared using ACS-grade sodium chloride (MP Biomedicals, Santa Ana, CA, USA; #194738) in sterile, endotoxin-free cell culture–grade water (Cytiva, Marlborough, MA, USA; #SH30529.02). Cells were centrifuged at 200 × g for 7 min at 4 °C (Sorvall ST8, Rotor 75005701) and washed in ice-cold PBS. The final pellet was resuspended in HBSS (Gibco; #14175-095) supplemented with 0.2% autologous serum and 10 mM HEPES (Corning, Corning, NY, USA; #25-060-CI) at 4 °C (henceforth referred to as HBSS+). Cell viability and concentration were assessed via trypan blue (0.4%; Gibco; #15250-061) exclusion using a Countess II FL automated cell counter (Thermo Scientific).

Neutrophils (1 × 10^6^ cells/mL in HBSS+, 1 × 10^6^ per well) were dispensed (100 μL) in a BioLite 96-well plate (Fisher) (n = 4 per condition). To standardize the final volume to 150 μL per well, negative control tissue culture plastic (TCP) wells received 40 μL HBSS+, and positive control TCP wells received 30 μL HBSS+ before cell addition. Heparin (Sigma–Aldrich, St. Louis, MO, USA; #H3393) was added to all wells at a final concentration of 10 U/mL to dissociate any NET-associated MPO (20, 78). Positive controls were stimulated with 100 nM phorbol 12-myristate 13-acetate (PMA; Sigma–Aldrich; #P8139); negative controls remained unstimulated. Plates were incubated at 37 °C in 5% CO_2_ for 3 or 6 h.

At each endpoint, plates were placed on ice for 10 min to arrest neutrophil activity prior to supernatant collection. Subsequently, 100 μL supernatant per well was transferred to 1.5 mL microcentrifuge tubes and centrifuged at 500 × g for 5 min at ambient temperature (Sorvall Legend XTR, Rotor 6133415; Thermo Scientific). A 50 μL aliquot of each clarified supernatant was transferred into clean tubes and stored at −20 °C until analysis (78).

### 2.2 Quantification of cytokine and degradative enzyme release

Supernatants were analyzed using a ProcartaPlex multiplex immunomagnetic assay (Thermo Fisher Scientific, Waltham, MA, USA). The assay panel quantified the following analytes: angiopoietin, fibroblast growth factor-2, granulocyte colony-stimulating factor (G-CSF), hepatocyte growth factor (HGF), interleukin (IL)-1β, IL-1 receptor antagonist, IL-6, IL-8, IL-10, IL-22, monocyte chemoattractant protein-1, matrix metalloproteinase-9 (MMP-9), myeloperoxidase (MPO), tumor necrosis factor-α, and vascular endothelial growth factor-A (VEGF-A).

MMP-9 and MPO were assayed using a 1:50 dilution in HBSS+ (Catalog Number: PPX-02-MXT2CHW, Lot: 463553-000). All other analytes were assayed using a 1:2 dilution in HBSS+ (Catalog Number: PPX-13-MXRWGXY, Lot: 450895-000). Of the 15 total analytes, MPO, MMP-9, IL-8, HGF, and VEGF-A were reliably above the assay’s lower limit of quantification (LLOQ). Analytes were excluded from the study if both sets of controls were below the assay’s LLOQ.

Assays were performed according to the manufacturer’s protocol. Briefly, standards were prepared via serial dilution to generate a multi-point calibration curve for each analyte. Samples, standards, and assay controls were run in duplicate. Plates were incubated with magnetic capture beads, detection antibodies, and streptavidin-phycoerythrin, and the unbound reagents were removed by washing on a magnetic plate washer. Then, bead-based fluorescence was quantified on a MAGPIX^®^ instrument (Luminex Corporation, Austin, TX, USA), and analyte concentrations were calculated using a 5-parameter logistic (5-PL) regression model in xPONENT^®^ software (Luminex). Only data that met assay acceptance criteria—bead count ≥50 events per analyte per well, and coefficient of variation (CV) ≤30% for replicates—were included in the final analysis. Values (for some unstimulated or treatment groups at 3 hours) below the LLOQ were recorded as half the lower limit of the standard range, which may introduce variance inflation and slight power reduction. Some values (for some stimulated groups for MPO) exceeded the upper limit of quantification (ULOQ) and were reassayed at a 1:100 dilution. All donors contributed to all conditions on the same multiplex plates, and each donor and time point used a separate 96-well plate for treatment during culture.

### 2.3 Statistical analysis and data visualization

All data were normalized in Microsoft Excel (Version 2405; Microsoft Corporation, Redmond, WA, USA) to each sample’s respective positive control mean, defined as 100% release for the corresponding analyte. This also addressed plate-to-plate variability, as each plate had its own positive and negative controls. Data normality was assessed using the Shapiro–Wilk test and inspection of Q–Q plots. Group differences were evaluated by one-way ANOVA with Holm–Šidák’s multiple comparisons test. Statistical analyses and data visualization were performed in GraphPad Prism (Version 8.4.3; GraphPad Software, San Diego, CA, USA) using a significance threshold of *p* < 0.05. Data are presented as box-and-whisker plots showing the median, interquartile range, and full range, with individual donor means overlaid as points.

*A priori* power analyses were conducted to confirm that sample sizes provided statistical power greater than 80%. As described above, all experimental and control groups were assayed in quadruplicate per plate across five independent experiments using blood from separate donors. Exclusion criteria were pre-established as a lack of statistical significance in the difference between positive (PMA-stimulated) and negative (untreated) MPO release levels, as PMA is a known stimulus for neutrophil MPO release (79). One donor met this criterion, and all data were discarded and re-assayed, as no significant differences between controls can mean either: high MPO in negative controls (indicating pre-existing inflammation or infection) or low MPO release from stimulated group neutrophils (indicating an issue with PMA stimulus) (23). Assumed effect sizes were based on previous experiments regarding primary neutrophil effector functions and HL60 cell model measurements of cytokine release (16, 21, 80). Power analyses were performed using the ‘pwr’ library in R (Version 4.3.0, R Foundation, Indianapolis, IN, USA) with a moderate assumed effect size due to prior investigations, α=0.05, and β=0.2. *Post-hoc* analysis was performed with a large effect size due to the observed differences in preliminary MPO data at 3 hours.

## 3 Results

### 3.1 MPO release

**Figure 2:**
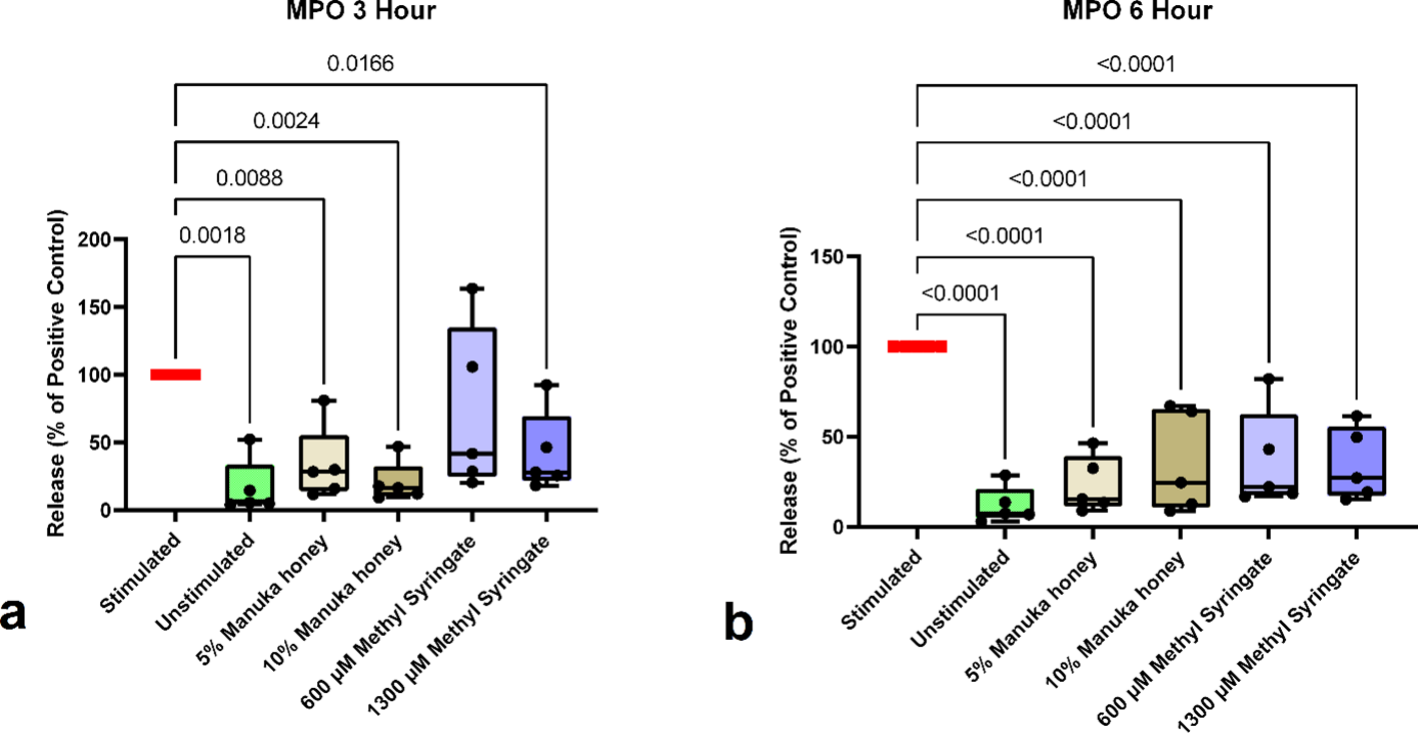
MPO release from neutrophils at 3-hour co-incubation **(a)** and 6-hour co-incubation **(b)**. Boxplots represent median and quartiles, whiskers represent range, and dots represent individual data sample values as percentages normalized to the mean of the individual donor control (n = 5). The red bar indicates positive PMA-stimulated neutrophil levels (100%), and green bars indicate untreated, unstimulated neutrophil levels.

The results from the MPO release study indicate that at the three-hour time point, all sample groups except for 600 µM methyl syringate statistically significantly reduced MPO levels in stimulated neutrophil supernatant, with the larger doses of both treatments (10% Manuka honey and 1300 µM methyl syringate) yielding the most considerable reductions **Table 1** (77.42% and 70.48% reductions from the positive control level, respectively) (**Figure 2a**). However, at the six-hour time point, all sample groups were significantly different from the positive control levels (**Figure 2b**). In contrast to the three-hour data, 5% Manuka honey and 1300 µM methyl syringate more consistently lowered MPO levels across donors (74.15% and 72.12%, respectively). These data suggest donor-to-donor variability in the efficacy of 600 µM at both time points, and 10% Manuka honey at later time points.

**Table 1:** Starting average concentrations of MPO in PMA-stimulated neutrophil culture supernatant (pg/mL).

Donor ID and treatment group	analyte level detected (pg/mL)
Donor 1–100 nM PMA 3 hour	3860928
Donor 2–100 nM PMA 3 hour	10739532
Donor 3–100 nM PMA 3 hour	4396238
Donor 4–100 nM PMA 3 hour	9695321
Donor 5–100 nM PMA 3 hour	18447795
Donor 1–100 nM PMA 6 hour	9324022
Donor 2–100 nM PMA 6 hour	15426543
Donor 3–100 nM PMA 6 hour	17658163
Donor 4–100 nM PMA 6 hour	22172905
Donor 5–100 nM PMA 6 hour	11306989

### 3.2 MMP-9 release

**Figure 3:**
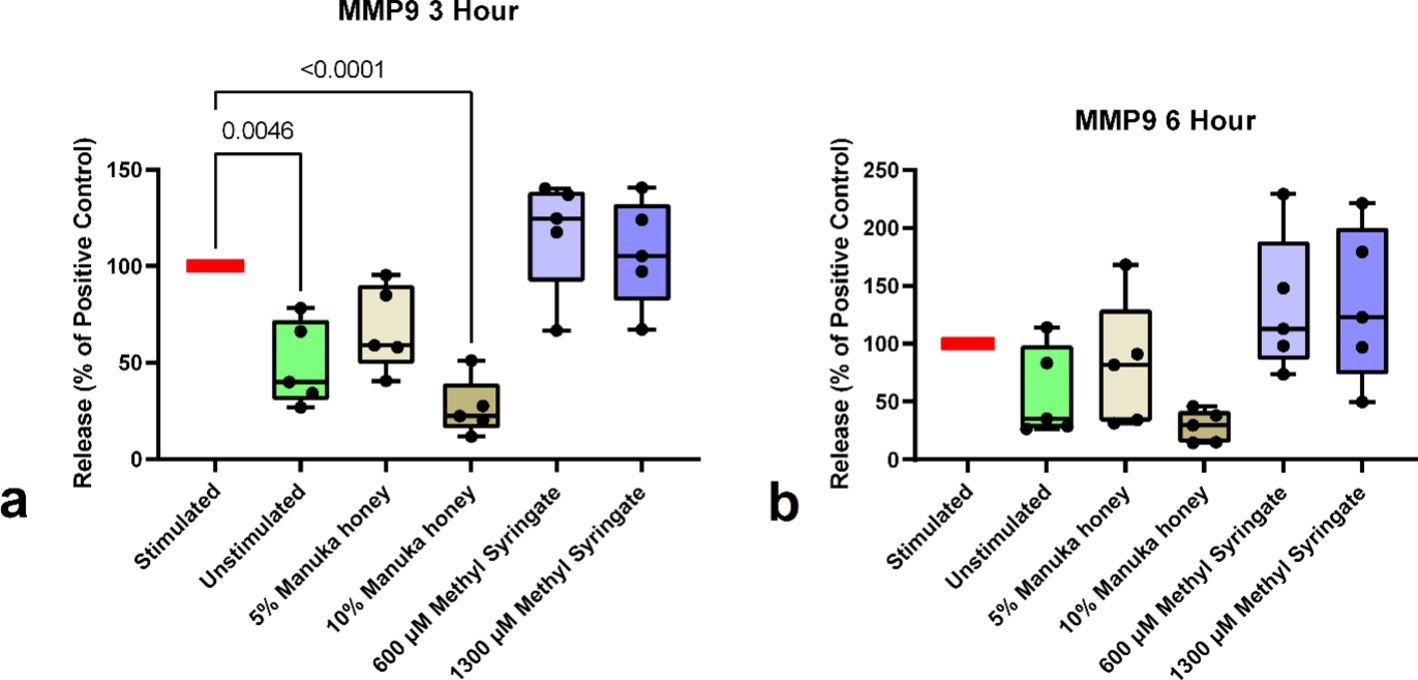
MMP-9 release from neutrophils at 3-hour co-incubation **(a)** and 6-hour co-incubation **(b)**. Boxplots represent median and quartiles, whiskers represent range, and dots represent individual data sample values as percentages normalized to the mean of the individual donor control (n = 5). The red bar indicates positive PMA-stimulated neutrophil levels (100%). The green bar indicates the level of untreated, unstimulated neutrophils.

MMP-9 release data showed statistically significant changes only with 10% Manuka honey at the three-hour time point, resulting in a 79.59% reduction compared with the positive control **Table 2** (**Figure 3a**). This statistical significance was not observed at the six-hour time point (**Figure 3b**). Of note, there is an apparent but slight increase in MMP-9 release from neutrophils in response to both concentrations of methyl syringate, although no statistical significance was observed.

**Table 2:** Starting average concentrations of MMP-9 in PMA-stimulated neutrophil culture supernatant (pg/mL).

Donor ID and treatment group	Analyte level detected (pg/mL)
Donor 1–100 nM PMA 3 hour	26920
Donor 2–100 nM PMA 3 hour	11995
Donor 3–100 nM PMA 3 hour	10737
Donor 4–100 nM PMA 3 hour	13919
Donor 5–100 nM PMA 3 hour	15281
Donor 1–100 nM PMA 6 hour	18466
Donor 2–100 nM PMA 6 hour	15328
Donor 3–100 nM PMA 6 hour	20329
Donor 4–100 nM PMA 6 hour	8933
Donor 5–100 nM PMA 6 hour	8683

### 3.3 IL-8 release

**Figure 4:**
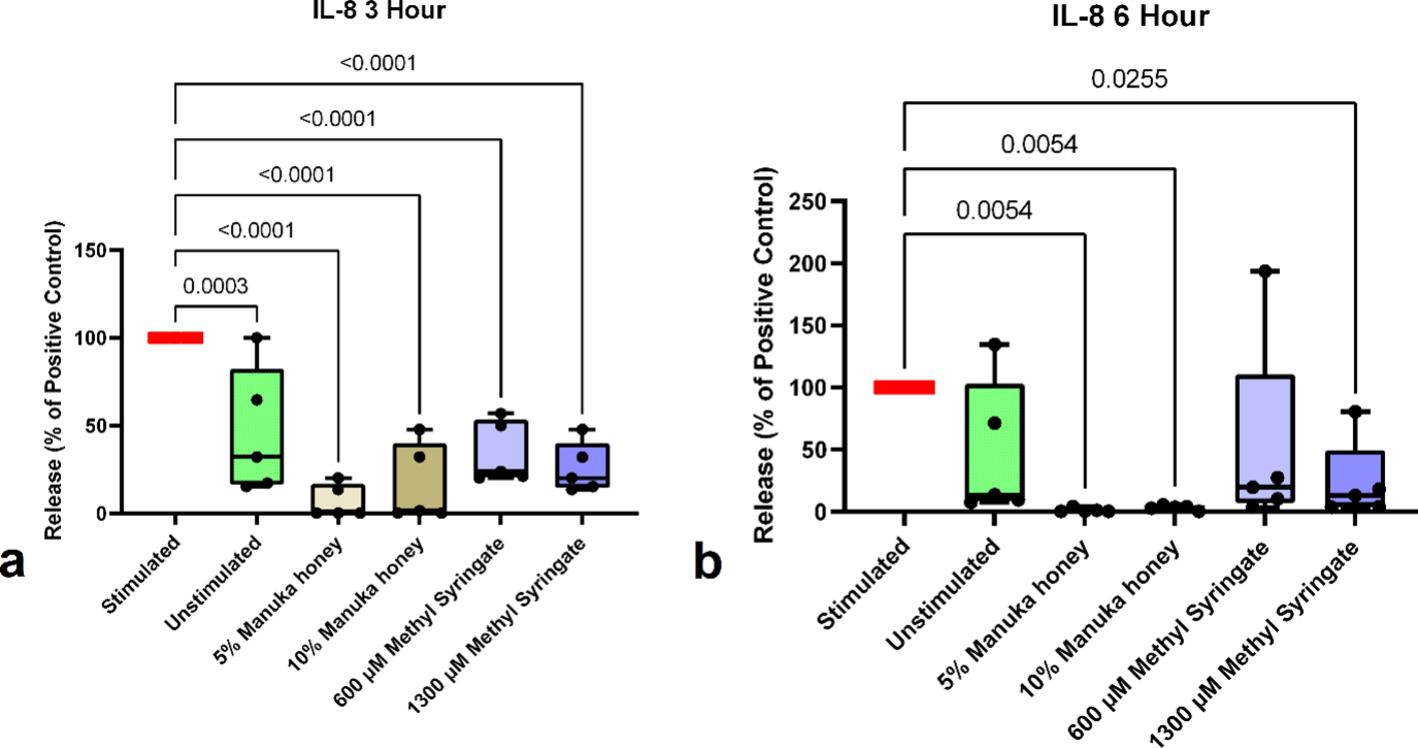
IL-8 release from neutrophils at 3-hour co-incubation **(a)** and 6-hour co-incubation **(b)**. Boxplots represent median and quartiles, whiskers represent range, and dots represent individual data sample values as percentages normalized to the mean of the individual donor control (n = 5). The red bar indicates positive PMA-stimulated neutrophil levels (100%). The green bar indicates the level of untreated, unstimulated neutrophils.

IL-8 values from this study indicate that at the three-hour timepoint, IL-8 is detectable in the culture media of PMA-stimulated neutrophils, and its release is reliably reduced by all treatment groups, demonstrating sharp reductions by 5% Manuka honey and 1300 µM methyl syringate (91.49% and 75.79%, respectively) (**Figure 4a**). At the six-hour time point, these effects were further pronounced, with IL-8 release being brought down to at or below unstimulated control levels. Manuka honey had remarkably high reductions in IL-8 release, with 5% and 10% reducing IL-8 by 98.51% and 95.99%, respectively. 600 µM methyl syringate reduced IL-8 release by 84.72%, and 1300 µM methyl syringate caused a 90.24% reduction (**Figure 4b**).

Despite donor-to-donor variation in baseline levels of PMA-stimulated IL-8 release (**Table 3**), the percentage of IL-8 reductions remained consistent across donors, with several Manuka honey treatment groups showing undetectable IL-8 levels.

**Table 3:** Starting average concentrations of IL-8 in PMA-stimulated neutrophil culture supernatant (pg/mL).

Donor ID and treatment group	Analyte level detected (pg/mL)
Donor 1 Stimulated 3 hour	22
Donor 2 Stimulated 3 hour	15
Donor 3 Stimulated 3 hour	121
Donor 4 Stimulated 3 hour	188
Donor 5 Stimulated 3 hour	121
Donor 1 Stimulated 6 hour	246
Donor 2 Stimulated 6 hour	81
Donor 3 Stimulated 6 hour	75
Donor 4 Stimulated 6 hour	54
Donor 5 Stimulated 6 hour	90

### 3.4 HGF release

**Figure 5:**
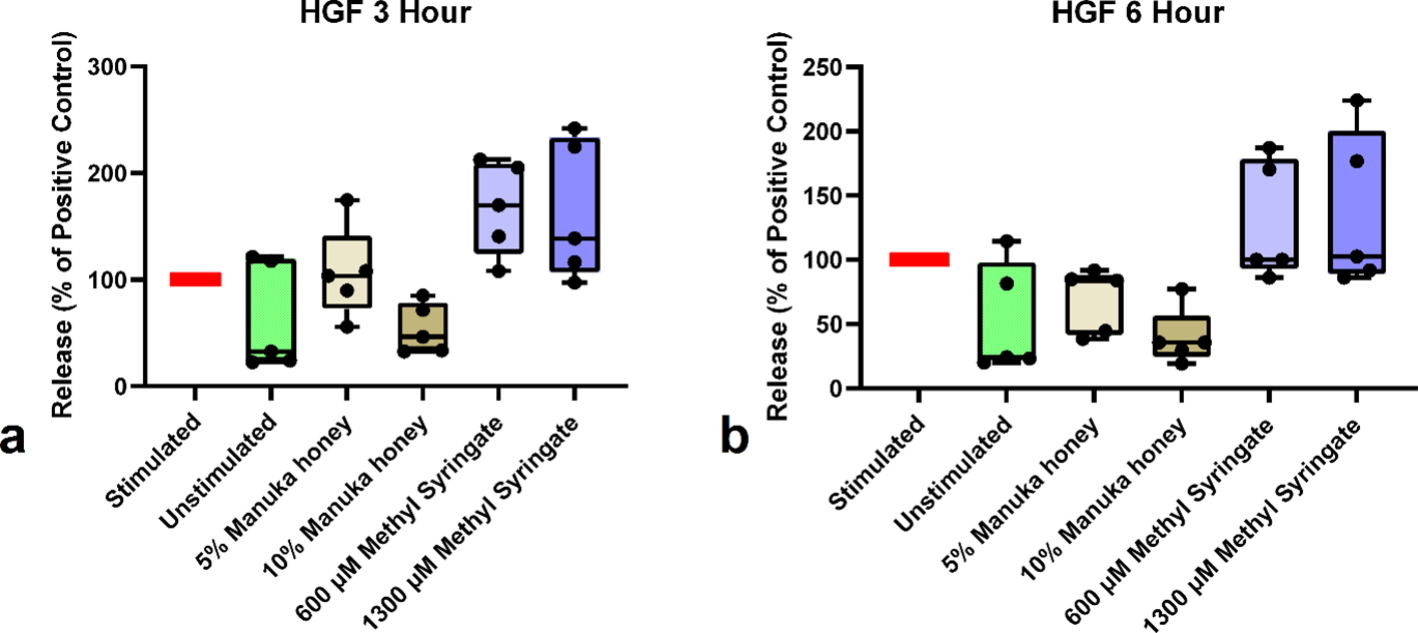
HGF release from neutrophils at 3-hour co-incubation **(a)** and 6-hour co-incubation **(b)**. Boxplots represent median and quartiles, whiskers represent range, and dots represent individual data sample values as percentages normalized to the mean of the individual donor control (n = 5). The red bar indicates positive PMA-stimulated neutrophil levels (100%). The green bar indicates the level of untreated, unstimulated neutrophils.

In contrast to previous findings, no significant differences in HGF release were detected between the sample groups at either the three- or six-hour time points (**Figures 5a, b**). These data indicate that neither Manuka honey nor methyl syringate inhibits neutrophil HGF release. As with the IL-8 and MMP-9 data, variance was observed in baseline levels of stimulated and unstimulated HGF release profiles (**Table 4**), including one donor whose unstimulated neutrophil levels of IL-8 and HGF were comparable to those of the stimulated control. However, these results were not found in MPO or MMP-9.

**Table 4:** Starting average concentrations of HGF in PMA-stimulated neutrophil culture supernatant (pg/mL).

Donor ID and treatment group	Analyte level detected (pg/mL)
Donor 1 Stimulated 3 hour	137
Donor 2 Stimulated 3 hour	126
Donor 3 Stimulated 3 hour	258
Donor 4 Stimulated 3 hour	277
Donor 5 Stimulated 3 hour	341
Donor 1 Stimulated 6 hour	332
Donor 2 Stimulated 6 hour	385
Donor 3 Stimulated 6 hour	137
Donor 4 Stimulated 6 hour	148
Donor 5 Stimulated 6 hour	385

### 3.5 VEGF-A release

**Figure 6:**
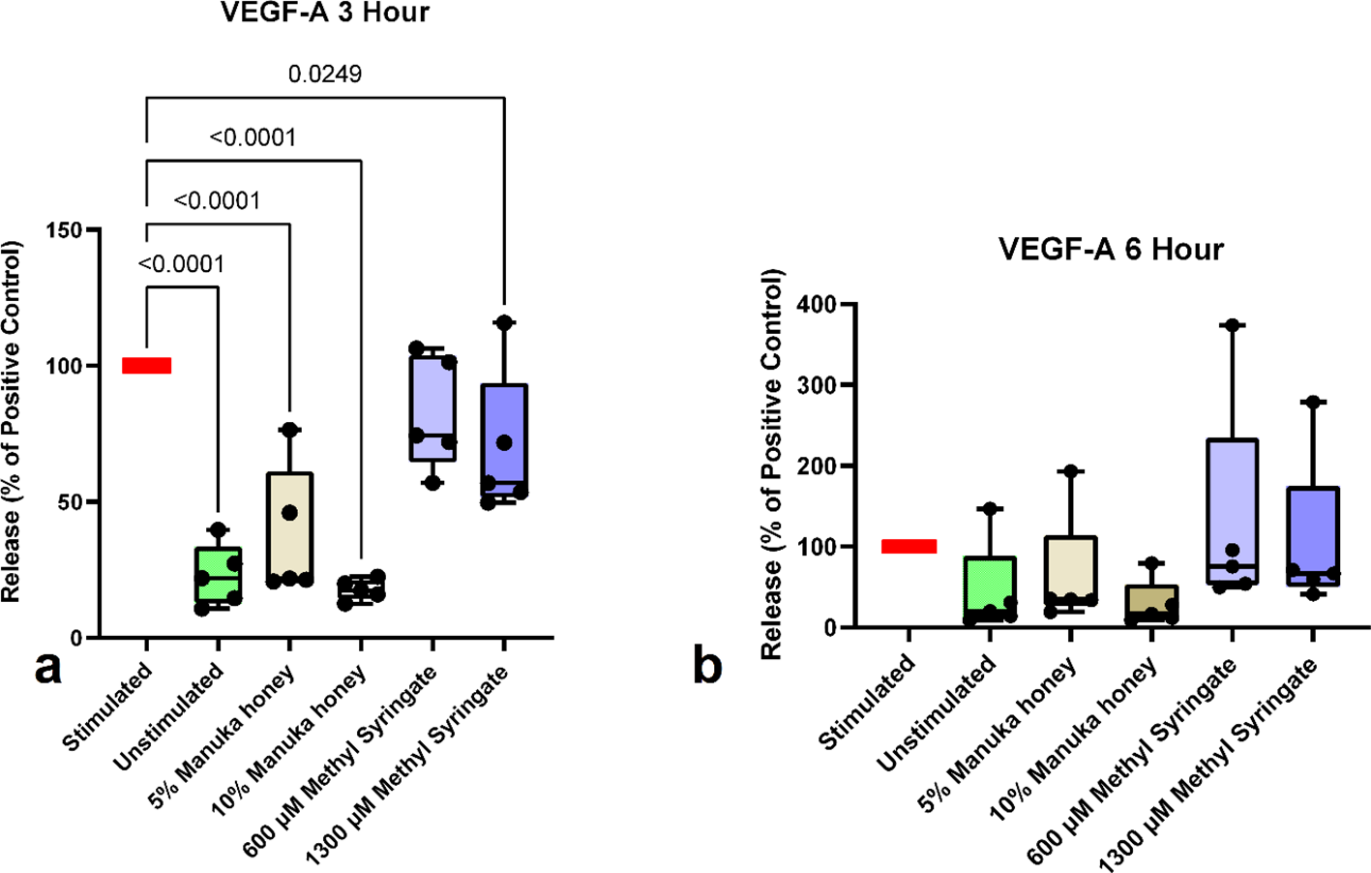
VEGF-A release from neutrophils at 3-hour co-incubation **(a)** and 6-hour co-incubation **(b)**. Boxplots represent median and quartiles, whiskers represent range, and dots represent individual data sample values as percentages normalized to the mean of the individual donor control (n = 5). The red bar indicates positive PMA-stimulated neutrophil levels (100%). The green bar indicates the level of untreated, unstimulated neutrophils.

Despite there being an insignificant change in neutrophil HGF release, VEGF-A release was impacted by the treatment of stimulated neutrophils with Manuka honey, but not methyl syringate, three hours after PMA stimulus **Table 5** (**Figure 6a**). 10% Manuka honey brought VEGF-A to baseline unstimulated levels (81.10% lower than positive control), while 5% Manuka honey reduced VEGF-A by only 58.68%. However, at the six-hour mark, statistically significant reductions were observed in both Manuka and methyl syringate concentrations (**Figure 6b**). Methyl syringate demonstrated a smaller effect, with 600 µM and 1300 µM reducing VEGF-A release by only 31.13% and 40.38%, respectively. Manuka honey had a much more dramatic effect on VEGF-A levels, reducing them by 69.23% at 5% Manuka honey and 83.65% at 10% Manuka honey.

**Table 5:** Starting average concentrations of VEGF-A in PMA-stimulated neutrophil culture supernatant (pg/mL).

Donor ID and treatment group	Analyte level detected (pg/mL)
Donor 1 Stimulated 3 hour	444
Donor 2 Stimulated 3 hour	395
Donor 3 Stimulated 3 hour	620
Donor 4 Stimulated 3 hour	535
Donor 5 Stimulated 3 hour	655
Donor 1 Stimulated 6 hour	346
Donor 2 Stimulated 6 hour	634
Donor 3 Stimulated 6 hour	860
Donor 4 Stimulated 6 hour	486
Donor 5 Stimulated 6 hour	107

## 4 Discussion

To date, the literature shows that in differentiated HL60-derived neutrophil models, Manuka honey reduces neutrophil superoxide production, chemotaxis, and NF-κB activation within a therapeutic range of 0.5–5% v/v. It was also observed that Manuka honey induced dose-dependent decreases in ROS activity and IκBα signaling when HL60s were co-cultured with it. Notably, chemotaxis was suppressed to baseline levels even at low honey concentrations, including under fMLP stimulation. Manuka honey also reduced pro-inflammatory cytokine secretion and degradative enzyme release, decreasing HL60 production of IL-1β, RANTES, and MIP-1α, as well as the ECM-degrading enzymes MMP-1 and MMP-9 (21, 80). Recently, reports of NET inhibition and intracellular reductions in ROS activity have been corroborated in a primary human neutrophil study. Additionally, the primary component driving these effects, methyl syringate, was elucidated (16). However, all investigations of neutrophil signaling and enzyme release in response to Manuka honey have been conducted using a neutrophil-like cell model.

While HL60 cells share some functional traits with primary neutrophils, such as increased phagocytic capacity, they retain notable deficiencies in pathways essential for fully mature neutrophil responses. A significant distinction lies in their respective roles in oxidative and inflammatory signaling. Primary neutrophils exhibit robust respiratory bursts, generating high levels of ROS. Although HL60 cells can initiate oxidative bursts, differences in NADPH oxidase component expression often result in reduced respiratory capacity compared with primary neutrophils (81). HL60 models also exhibit reduced chemotactic precision and less efficient degranulation than primary neutrophils, especially in dynamic environments (82). Altered intracellular signaling further contributes to functional discrepancies. For example, studies using Akt inhibitors have shown that HL60 cells exhibit weaker Akt-dependent signaling than primary neutrophils, resulting in impaired chemotaxis and attenuated activation responses (83). In addition, primary neutrophils exhibit more finely regulated lifespan and apoptotic pathways, whereas HL60 cells often fail to fully recapitulate the physiological turnover of mature neutrophils (84). Thus, while HL60 cell models are highly useful for modeling neutrophil behavior and provide a crucial validation step for further investigation, they are not a one-for-one match for primary human neutrophil effector functions or signaling.

Thus far, Manuka honey and methyl syringate are potent modulators of neutrophil effector functions (16). However, the previous study did not investigate downstream cytokine, enzyme, and growth factor release, leaving a critical gap in understanding the mechanisms underlying the immunomodulatory capabilities of Manuka honey and methyl syringate. This literature gap has also led to the absence of a starting point for mechanistic pathway investigations seeking to elucidate how Manuka honey and methyl syringate act on neutrophils to prevent inflammatory behaviors. Therefore, this investigation has yielded two significant findings. First, and most directly, it has established a release profile for PMA-activated primary human neutrophils treated with Manuka honey and methyl syringate. Second, from the release profile, this study highlights a smaller set of neutrophil membrane receptors that may potentially be upstream mechanisms by which Manuka honey and its constituent, methyl syringate, reduce neutrophil inflammation.

The data presented in this investigation further corroborates previous findings that Manuka honey and methyl syringate inhibit NETosis. MPO, released by neutrophils during NETosis, has been used as a marker for NETs in prior investigations (78). This study observed a similar reduction of MPO concentrations in the supernatant of PMA-activated neutrophils in response to Manuka honey and methyl syringate. The percent reductions in extracellular MPO by 5% and 10% Manuka honey, and 600 µM and 1300 µM methyl syringate are remarkably similar to the magnitude of decreases in NET-associated DNA via Sytox Orange cell staining in previous literature (16).

Regarding MMP-9 release, the findings of this study are somewhat consistent with those from previous studies using HL60 cells. Manuka honey inhibited MMP-9 release in primary human neutrophils, although to a much lesser extent than in HL60 Cells, for which reports claimed inhibition at or below detection levels. Interestingly, while Manuka honey inhibited MMP-9 release at the three-hour time point, no such inhibition was observed in the methyl syringate groups. Thus, it may be concluded that the ability of Manuka honey to inhibit MMP-9 release in both HL60 cells and primary human neutrophils is not methyl syringate-mediated, unlike Manuka honey’s capacity for NET and ROS activity inhibition. These data, along with others reported in this study, suggest key functional differences between whole Manuka honey and methyl syringate. These differences provide insight into which therapeutic compound is likely more suited to specific applications. If the remodeling capacity of neutrophils needs to be preserved to support neovascularization, a case can be made for using methyl syringate alone rather than Manuka honey, as it does not strongly suppress MMP-9 release.

IL-8 release, in sharp contrast to HL60 data, was strongly inhibited by Manuka honey and methyl syringate in primary human neutrophils. Prior reports using HL60 cell lines indicated that lower concentrations (0.5% and 3%) of Manuka honey may stimulate IL-8 release, whereas this study showed that higher concentrations (5% and 10%) inhibit IL-8 release (20). Additionally, inflammatory markers at the 10% Manuka honey concentration conflict with previously established cytotoxic limits (19, 21). Overall, the combination of MPO and IL-8 data further emphasizes Manuka honey’s potential as an anti-inflammatory bioactive compound, reinforces recent findings that methyl syringate is likely responsible for many of these immunomodulatory effects, and demonstrates strong potential on its own as a therapeutic molecule. Additionally, the findings regarding MPO and IL-8 inhibition are exciting, as MPO is a key cytotoxic enzyme found in many pathologies with dysregulated neutrophil responses, and IL-8 drives excessive neutrophil “swarming” to sites of inflammation. By regulating both neutrophil release factors, Manuka honey and methyl syringate directly mitigate effector functions *in situ* and prevent the positive feedback loop of pro-inflammatory signaling that drives a vicious cycle of sustained acute inflammation (3, 48).

While excessive neutrophil inflammation presents an opportunity for therapeutic interventions, neutrophils are increasingly recognized as vital players in the tissue remodeling that occurs after (and due to) acute inflammation at the affected site (56). Upon activation and chemotaxis to the site of injury, infection, or implantation, neutrophils initiate the inflammatory response to clear pathogens, then the cleanup phase to phagocytose debris, and finally release growth factors and matrix-remodeling enzymes to drive functional tissue regeneration (4, 55, 85). A key question left unanswered by investigations thus far is whether inhibiting neutrophil inflammation would inadvertently compromise their regenerative capacity. The findings of this study are mixed in this regard. HGF release appeared to be unaffected by Manuka honey and, in the case of methyl syringate, potentially enhanced, although not statistically significantly. VEGF-A release, however, was lowered by Manuka honey at both three and six-hour time points, and at the six-hour time point only for methyl syringate. These data indicate that neutrophil inflammatory behaviors are not strictly linked to HGF release and that neutrophil HGF secretion can potentially be preserved even with treatment with Manuka honey and methyl syringate. As with MMP-9 data, VEGF-A release is another parameter that distinguishes Manuka honey from its phenolic component alone, methyl syringate. At three hours, VEGF-A release was nearly completely diminished by treatment with Manuka honey, while neither methyl syringate concentrations did not statistically significantly impact VEGF-A release levels. Qualitatively, while the VEGF-A release eventually declined from that of the PMA-stimulated control level at six hours in both methyl syringate concentrations, the decrease was less than that of the Manuka honey sample groups. Manuka honey consistently reduced VEGF-A release to levels comparable to those of unstimulated cells, whereas, even at 1300 µM and 6 hours after treatment, methyl syringate reduced VEGF-A levels by only 40%. These findings are critical for distinguishing the potential applications and contexts of Manuka honey and methyl syringate, as MMP-9 and VEGF-A are both factors that drive neutrophil-mediated angiogenesis and tissue revascularization, both of which are critical for proper wound healing (67). While previous literature has been focused on methyl syringate as a key component of Manuka honey, this study presents evidence that differentiates methyl syringate as an individual therapeutic, with different benefits than those of whole Manuka honey, such as the partial preservation of VEGF-A release, and no strong effect on MMP-9 levels.

This investigation presented evidence of the efficacy of Manuka honey and methyl syringate to reduce neutrophil inflammatory marker release, while wholly or partially preserving factors related to angiogenesis and healing. However, there are some key limitations to the present study.

Of note, baseline levels of cytokines, growth factors, and enzyme release among donors were highly variable, especially for HGF, VEGF-A, and IL-8. Based on the existing literature, this is to be expected, with widely variable levels of the mentioned analytes, even within a single assay, and in serum (86–88). Given that neutrophils themselves are notoriously heterogeneous, this likely compounds the variability in cytokine levels observed among donors in this study. Therefore, normalization to each donor’s positive control was necessary, as baseline levels were highly variable, yet the data trends remained consistent across donors. Due to this variability, the normalization methods and power analysis may require further in-depth study with a large donor pool to detect more modest trends, such as those observed in the MMP-9 data. Additionally, normality testing, ANOVA, and *post-hoc* Holm-Šídák were applied to each analyte and each timepoint after normalization to the donor-positive control, which increased the risk of false positives. Despite this, the effect sizes and low p-values seen from the data make it unlikely that a type 1 error occurred. Overall, these limitations highlight the need for broad-ranging clinical studies involving neutrophil heterogeneity, especially in response to neutrophil-targeted drugs and therapeutics. This study sought to highlight the potential of Manuka honey and methyl syringate to reduce specific inflammatory cytokines and chemokines while preserving pro-regenerative neutrophil release profiles. However, for these to be translated into the clinic, large-scale population studies should be conducted.

Other key limitations of this investigation are related to confounding variables that whole Manuka honey introduces. Namely, high osmolarity and low pH, which have traditionally been attributed as factors that enhance Manuka honey’s antibacterial properties but were not controlled in this study, to investigate the benefits and drawbacks of whole Manuka honey versus isolated methyl syringate (89). However, these factors can complicate the assessment of Manuka honey as an anti-inflammatory compound. The pH of the local microenvironment plays a pivotal role in shaping neutrophil activity and inflammatory outcomes. Manuka honey has a moderately acidic pH (3.5–4.5), which may contribute to its ability to modulate neutrophil-mediated responses (90). Acidic conditions have been shown to enhance neutrophil antimicrobial function and increase cytokine and chemokine production, thereby affecting both the magnitude and duration of the inflammatory response (91, 92). However, the findings of this study indicate that although Manuka honey creates an acidic environment, the ensuing cytokine and chemokine release remains limited. This effect further suggests that the activity of the phenolic and antioxidant molecules within Manuka honey is responsible for reductions in neutrophil inflammation. In contrast, honey-induced acidification may also limit excessive proteolytic activity at sites of tissue injury, preserving extracellular matrix structure and supporting coordinated healing responses (93).

In addition to pH, neutrophil function is highly sensitive to osmotic changes within the local microenvironment. Alterations in osmotic pressure can initiate intracellular signaling pathways that influence neutrophil activation and effector responses. Elevated osmolarity has been shown to modulate intracellular calcium flux and activate downstream signaling cascades that promote the release of pro-inflammatory mediators, thereby potentially exacerbating inflammatory responses (94, 95). Osmotic conditions may also affect chemotactic signaling, as neutrophil responsiveness to IL-8 depends on receptor–ligand interactions that can be altered by changes in receptor sensitivity across varying osmotic environments (96). Based on the data collected in this investigation, it is suggested that the bioactivity of Manuka honey may help counteract the potential neutrophil-inducing effects of pH and osmotic conditions.

Additionally, this investigation used concentration ranges found to be beneficial outside the context of biomaterial incorporation. While Manuka honey concentration ranges from 0.1-10% have been incorporated into biomaterials, the amount of Manuka honey eluted was variable, and after 21 days, it cumulated to 20.4 mg/mL (about 2% w/v) (97). It is important to note that while the 5% and 10% Manuka honey concentrations were above what was eluted in this recent study, the methyl syringate concentrations fell within a range that would be found in a 2% Manuka honey elution (15).

While this study presented evidence that Manuka honey and methyl syringate can enhance the resolution of acute-phase inflammation, it was limited in scope by several factors. First, it was performed *in vitro* and examined only neutrophil behavior. The host response to injury and biomaterial implantation is a multifaceted, complex, and interwoven process involving numerous cell types (98). To truly determine the feasibility of Manuka honey and methyl syringate as therapeutics, co-culture studies and, eventually, *in vivo* studies should be performed to identify which other cell types are involved in the phenomena discussed in this paper and whether they are sustained in a dynamic, multicellular environment. As neutrophils are short-lived, especially *ex vivo*, timepoints were limited to short-term (3 and 6 hours). These constraints are of note, especially for promoting angiogenesis and functional tissue regeneration, as this study used surrogate markers known to be released by neutrophils. Further studies should be conducted to examine how these surrogate signals interact within the multicellular, interplay milieu of the target site for potential therapeutic application, such as the host-biomaterial interface of a Manuka honey or methyl syringate-laden implant. It would be especially important to investigate the interplay among macrophages, fibroblasts, and endothelial cells in the context of Manuka honey or methyl syringate treatment. Additionally, this study examined released signals, enzymes, and growth factors, but not the signaling pathways that drive their release. However, cellular signaling pathways are notoriously intricate and multifaceted, making it daunting to identify a single pathway, let alone a few, especially in neutrophil biology, where much remains to be learned. In this endeavor, it is paramount to have a starting point. Based on the investigation’s findings, likely pathways of inhibition or alteration responsible for the immunomodulatory capabilities of Manuka honey and methyl syringate are hypothesized for further mechanistic studies.

For example, toll-like receptor 4 (TLR4) activation and protein kinase-C (PKC) signaling interact to regulate neutrophil oxidative responses, inflammatory mediator release, and NET release. Upon stimulation, such as by bacterial membrane lipopolysaccharide (LPS) or PMA, PKC isoforms (notably PKC-α, PKC-β, and PKC-δ) phosphorylate the p47phox subunit of nicotinamide adenine dinucleotide phosphate (NADPH) oxidase, enabling its translocation to the membrane and initiating superoxide production during neutrophil respiratory burst (99–101). TLR4 engagement enhances this process, with PKC-δ contributing to TLR4 upregulation and strengthening downstream signaling in response to pro-inflammatory stimuli (102, 103). NADPH oxidase–derived ROS amplify TLR4 signaling, creating a feed-forward loop that intensifies neutrophil activation and the release of inflammatory mediators, including MPO, IL-8, and VEGF-A (104, 105). IL-8 generated downstream of TLR4 promotes neutrophil chemotaxis and survival, while VEGF-A release contributes to vascular permeability and the recruitment of additional immune cells. PKC, in concert with PLC and PI3K, further couples TLR4 activation to NADPH oxidase assembly (106). Together, the PKC–NADPH oxidase-TLR4 axis orchestrates key antimicrobial and inflammatory functions in neutrophils, but excessive activation can exacerbate tissue injury. In disease settings such as diabetic nephropathy, inhibiting NADPH oxidase mitigates oxidative damage through PKC-dependent pathways (107). Based on the findings of the present study and previous literature, it is hypothesized that this signaling axis may likely be involved. Thus, inhibitor and signaling protein phosphorylation studies are currently underway to investigate the mechanisms behind methyl syringate and Manuka honey treatment and the reduction in pro-inflammatory signaling and effector functions in neutrophils.

Overall, the goal of these investigations is to develop a biomaterial additive or a localized therapeutic to reduce excessive neutrophil inflammation within a specific environment. While the *in vitro* response from isolated neutrophils shows tremendous promise, further studies are needed to assess the ability of these molecules to incorporate into biomaterials or wound dressings. As this study was performed *ex vivo* and did not incorporate biomaterials, further studies are needed to connect the promise of this study to its feasibility for clinical use. Therefore, further studies should be conducted to characterize the release, bioavailability, and effects on material properties resulting from incorporating Manuka honey/methyl syringate into biomaterials. This is a critical step in translating benchtop results to the clinic, as processing, storage, and implantation sites can significantly affect the bioactivity of these materials. While Manuka honey has been used in wound-healing studies and, more recently, in tissue-engineering approaches, methyl syringate has not been studied or characterized in this way. The novelty of this polyphenol as a neutrophil therapeutic presents unique challenges, as until recently, the majority of research has been largely food-science related, with very few biomedical applications (108).

## 5 Conclusion

In summary, this study’s findings suggest that Manuka honey and its primary phenolic component, methyl syringate, reduce neutrophil pro-inflammatory signaling. Furthermore, the data presented in this investigation indicate potential key functional differences between whole Manuka honey and methyl syringate, specifically regarding the preservation of growth factors and pro-remodeling matrix metalloproteinases associated with functional wound regeneration release despite reductions in pro-inflammatory signaling and effector functions. Thus, the possibility of ameliorating excessive neutrophil inflammation while retaining the wound-healing aspects of neutrophil activation may yet be possible.

## Data Availability

The raw data supporting the conclusions of this article will be made available by the authors, without undue reservation.

## References

[1] Anderson JM Rodriguez A Chang DT . Foreign body reaction to biomaterials. Semin Immunol. (2008) 20:86–100. doi: 10.1016/j.smim.2007.11.004, PMID: 18162407 PMC2327202

[2] Fetz AE Fantaziu CA Smith RA Radic MZ Bowlin GL . Surface area to volume ratio of electrospun polydioxanone templates regulates the adsorption of soluble proteins from human serum. Bioengineering. (2019) 6:78. doi: 10.3390/bioengineering6030078, PMID: 31480458 PMC6784194

[3] Selders GS Fetz AE Radic MZ Bowlin GL . An overview of the role of neutrophils in innate immunity, inflammation and host-biomaterial integration. Regenerative biomaterials. (2017) 4:55–68. doi: 10.1093/rb/rbw041, PMID: 28149530 PMC5274707

[4] Wilgus TA Roy S McDaniel JC . Neutrophils and wound repair: positive actions and negative reactions. Adv Wound Care. (2013) 2:379–88. doi: 10.1089/wound.2012.0383, PMID: 24527354 PMC3763227

[5] Sabbatini M Magnelli V Renò F . NETosis in wound healing: when enough is enough. Cells. (2021) 10:494. doi: 10.3390/cells10030494, PMID: 33668924 PMC7996535

[6] Bouchery T Harris N . Neutrophil–macrophage cooperation and its impact on tissue repair. Immunol Cell Biol. (2019) 97:289–98. doi: 10.1111/imcb.12241, PMID: 30710448

[7] Chrysanthopoulou A Mitroulis I Apostolidou E Arelaki S Mikroulis D Konstantinidis T . Neutrophil extracellular traps promote differentiation and function of fibroblasts. J pathology. (2014) 233:294–307. doi: 10.1002/path.4359, PMID: 24740698

[8] Cassatella MA . The neutrophil: an emerging regulator of inflammatory and immune response Vol. 83. Basel, Switzerland: Karger Medical and Scientific Publishers (2003).

[9] Fialkow L Wang Y Downey GP . Reactive oxygen and nitrogen species as signaling molecules regulating neutrophil function. Free Radical Biol Med. (2007) 42:153–64. doi: 10.1016/j.freeradbiomed.2006.09.030, PMID: 17189821

[10] Jaeschke H Bautista AP Spolarics Z Spitzer JJ . Superoxide generation by neutrophils and Kupffer cells during *in vivo* reperfusion after hepatic ischemia in rats. J leukocyte Biol. (1992) 52:377–82. doi: 10.1002/jlb.52.4.377, PMID: 1328439

[11] Jaganjac M Cipak A Schaur RJ Zarkovic N . Pathophysiology of neutrophil-mediated extracellular redox reactions. Front Biosci (Landmark Ed). (2016) 21:839–55. doi: 10.2741/4423, PMID: 26709808

[12] Luo HR Loison F . Constitutive neutrophil apoptosis: mechanisms and regulation. Am J hematol. (2008) 83:288–95. doi: 10.1002/ajh.21078, PMID: 17924549

[13] Blackwell TS Blackwell TR Holden EP Christman BW Christman JW . *In vivo* antioxidant treatment suppresses nuclear factor-kappa B activation and neutrophilic lung inflammation. J Immunol. (1996) 157:1630–7. doi: 10.4049/jimmunol.157.4.1630, PMID: 8759749

[14] Stoiber W Obermayer A Steinbacher P Krautgartner W-D . The role of reactive oxygen species (ROS) in the formation of extracellular traps (ETs) in humans. Biomolecules. (2015) 5:702–23. doi: 10.3390/biom5020702, PMID: 25946076 PMC4496692

[15] Main EN Bowlin GL . Potential for Manuka honey-inspired therapeutics to improve the host–biomaterial response. MedComm–Biomaterials Appl. (2022) 1:e18. doi: 10.1002/mba2.18, PMID: 41744314

[16] Main EN Huang JC Bowlin GL . Methyl syringate: A primary driving factor in manuka honeys ability to ameliorate neutrophil intracellular ROS activity and NETosis. Front Bioscience-Landmark. (2024) 29:255. doi: 10.31083/j.fbl2907255, PMID: 39082351 PMC11973827

[17] Minden-Birkenmaier BA Bowlin GL . Honey-based templates in wound healing and tissue engineering. Bioengineering. (2018) 5:46. doi: 10.3390/bioengineering502004, PMID: 29903998 PMC6027142

[18] Minden-Birkenmaier BA . Manuka honey as a tissue engineering bioactive: effect on neutrophil inflammatory behavior. Memphis, TN, USA: The University of Memphis (2020).

[19] Minden-Birkenmaier BA Smith RA Radic MZ van der Merwe M Bowlin GL . Manuka honey reduces NETosis on an electrospun template within a therapeutic window. Polymers. (2020) 12:1430. doi: 10.3390/polym12061430, PMID: 32604824 PMC7362002

[20] Minden-Birkenmaier B Meadows M Cherukuri K Smeltzer MP Radic M Bowlin G . The Effect of Manuka Honey on dHl-60 Cytokine, Chemokine, and Matrix-Degrading Enzyme Release under Inflammatory Conditions. Med One. (2019) 4:e190005. doi: 10.20900/mo.20190005, PMID: 31245627 PMC6594701

[21] Minden-Birkenmaier BA Cherukuri K Smith RA Radic MZ Bowlin GL . Manuka honey modulates the inflammatory behavior of a dHL-60 neutrophil model under the cytotoxic limit. Int J biomaterials. (2019) 2019:1687–8787. doi: 10.1155/2019/6132581, PMID: 30936919 PMC6415307

[22] Malle E Furtmüller P Sattler W Obinger C . Myeloperoxidase: a target for new drug development? Br J Pharmacol. (2007) 152:838–54. doi: 10.1038/sj.bjp.0707358, PMID: 17592500 PMC2078229

[23] Lin W Chen H Chen X Guo C . The roles of neutrophil-derived myeloperoxidase (MPO) in diseases: the new progress. Antioxidants. (2024) 13:132. doi: 10.3390/antiox13010132, PMID: 38275657 PMC10812636

[24] Davies MJ Hawkins CL . The role of myeloperoxidase in biomolecule modification, chronic inflammation, and disease. Antioxidants Redox Signaling. (2020) 32:957–81. doi: 10.1089/ars.2020.8030, PMID: 31989833

[25] El Kebir D Joózsef L Pan W JnG F . Myeloperoxidase delays neutrophil apoptosis through CD11b/CD18 integrins and prolongs inflammation. Circ Res. (2008) 103:352–9. doi: 10.1161/01.RES.0000326772.76822.7a, PMID: 18617697

[26] Haegens A Vernooy JH Heeringa P Mossman BT Wouters EF . Myeloperoxidase modulates lung epithelial responses to pro-inflammatory agents. Eur Respir J. (2008) 31:252–60. doi: 10.1183/09031936.00029307, PMID: 18057061

[27] Lau D Mollnau H Eiserich JP Freeman BA Daiber A Gehling UM . Myeloperoxidase mediates neutrophil activation by association with CD11b/CD18 integrins. Proc Natl Acad Sci. (2005) 102:431–6. doi: 10.1073/pnas.0405193102, PMID: 15625114 PMC544285

[28] Tseng A Kim K Li J Cho J . Myeloperoxidase negatively regulates neutrophil–endothelial cell interactions by impairing αMβ2 integrin function in sterile inflammation. Front Med. (2018) 5:134. doi: 10.3389/fmed.2018.00134, PMID: 29780806 PMC5946029

[29] Yang J Cheng Y Ji R Zhang C . Novel model of inflammatory neointima formation reveals a potential role of myeloperoxidase in neointimal hyperplasia. Am J Physiology-Heart Circulatory Physiol. (2006) 291:H3087–93. doi: 10.1152/ajpheart.00412.2006, PMID: 16844918

[30] Sexton TR Wallace EL Macaulay TE Charnigo RJ Evangelista V Campbell CL . The effect of rosuvastatin on thromboinflammation in the setting of acute coronary syndrome. J Thromb Thrombolysis. (2015) 39:186–95. doi: 10.1007/s11239-014-1142-x, PMID: 25307674 PMC4320305

[31] Khalilova IS Dickerhof N Mocatta TJ Bhagra CJ McClean DR Obinger C . A myeloperoxidase precursor, pro-myeloperoxidase, is present in human plasma and elevated in cardiovascular disease patients. PloS One. (2018) 13:e0192952. doi: 10.1371/journal.pone.0192952, PMID: 29590135 PMC5873943

[32] Patel AA Ginhoux F Yona S . Monocytes, macrophages, dendritic cells and neutrophils: an update on lifespan kinetics in health and disease. Immunology. (2021) 163:250–61. doi: 10.1111/imm.13320, PMID: 33555612 PMC8207393

[33] Sumagin R Finkielsztein A Slater T Mascarenhas LL Mehl L Butin-Israeli V . Neutrophil microparticles deliver active myeloperoxidase to injured mucosa to inhibit epithelial wound healing. FASEB J. (2017) 31:465.467–465.467. doi: 10.1096/fasebj.31.1_supplement.465.7, PMID: 28242649 PMC5360559

[34] Nguyen‐Chi M Luz‐Crawford P Balas L Sipka T Contreras‐López R Barthelaix A . Pro-resolving mediator protectin D1 promotes epimorphic regeneration by controlling immune cell function in vertebrates. Br J Pharmacol. (2020) 177:4055–73. doi: 10.1111/bph.15156, PMID: 32520398 PMC7429485

[35] Yang W Tao Y Wu Y Zhao X Ye W Zhao D . Neutrophils promote the development of reparative macrophages mediated by ROS to orchestrate liver repair. Nat Commun. (2019) 10:1076. doi: 10.1038/s41467-019-09046-8, PMID: 30842418 PMC6403250

[36] Yoon H-K Cho H-Y Kleeberger SR . Protective role of matrix metalloproteinase-9 in ozone-induced airway inflammation. Environ Health perspectives. (2007) 115:1557–63. doi: 10.1289/ehp.10289, PMID: 18007984 PMC2072825

[37] Lin M Jackson P Tester AM Diaconu E Overall CM Blalock JE . Matrix metalloproteinase-8 facilitates neutrophil migration through the corneal stromal matrix by collagen degradation and production of the chemotactic peptide Pro-Gly-Pro. Am J pathology. (2008) 173:144–53. doi: 10.2353/ajpath.2008.080081, PMID: 18556780 PMC2438292

[38] Koymans KJ Bisschop A Vughs MM Van Kessel KP De Haas CJ Van Strijp JA . Staphylococcal superantigen-like protein 1 and 5 (SSL1 & SSL5) limit neutrophil chemotaxis and migration through MMP-inhibition. Int J Mol Sci. (2016) 17:1072. doi: 10.3390/ijms17071072, PMID: 27399672 PMC4964448

[39] Chuah C Jones MK Burke ML McManus DP Owen HC Gobert GN . Defining a pro-inflammatory neutrophil phenotype in response to schistosome eggs. Cell Microbiol. (2014) 16:1666–77. doi: 10.1111/cmi.12316, PMID: 24898449

[40] Marshall DC Lyman SK McCauley S Kovalenko M Spangler R Liu C . Selective allosteric inhibition of MMP9 is efficacious in preclinical models of ulcerative colitis and colorectal cancer. PloS One. (2015) 10:e0127063. doi: 10.1371/journal.pone.0127063, PMID: 25961845 PMC4427291

[41] Sagel SD Kapsner RK Osberg I . Induced sputum matrix metalloproteinase-9 correlates with lung function and airway inflammation in children with cystic fibrosis. Pediatr pulmonol. (2005) 39:224–32. doi: 10.1002/ppul.20165, PMID: 15635615

[42] Yoshihara Y Nakamura H Obata K Yamada H Hayakawa T Fujikawa K . Matrix metalloproteinases and tissue inhibitors of metalloproteinases in synovial fluids from patients with rheumatoid arthritis or osteoarthritis. Ann rheumatic dis. (2000) 59:455–61. doi: 10.1136/ard.59.6.455, PMID: 10834863 PMC1753174

[43] Wang H Gao M Li J Sun J Wu R Han D . MMP-9-positive neutrophils are essential for establishing profibrotic microenvironment in the obstructed kidney of UUO mice. Acta Physiologica. (2019) 227:e13317. doi: 10.1111/apha.13317, PMID: 31132220

[44] Thirumangalakudi L Samany PG Owoso A Wiskar B Grammas P . Angiogenic proteins are expressed by brain blood vessels in Alzheimer’s disease. J Alzheimer’s Dis. (2006) 10:111–8. doi: 10.3233/JAD-2006-10114, PMID: 16988487

[45] Tessem JS Jensen JN Pelli H Dai XM Zong XH Stanley ER . Critical roles for macrophages in islet angiogenesis and maintenance during pancreatic degeneration. Diabetes. (2008) 57:1605–17. doi: 10.2337/db07-1577, PMID: 18375440 PMC2575065

[46] Murphy PM Tiffany HL . Cloning of complementary DNA encoding a functional human interleukin-8 receptor. Science. (1991) 253:1280–3. doi: 10.1126/science.1891716, PMID: 1891716

[47] Hu N Westra J Rutgers A Doornbos-Van der Meer B Huitema MG Stegeman CA . Decreased CXCR1 and CXCR2 expression on neutrophils in anti-neutrophil cytoplasmic autoantibody-associated vasculitides potentially increases neutrophil adhesion and impairs migration. Arthritis Res Ther. (2011) 13:R201. doi: 10.1186/ar3534, PMID: 22152684 PMC3334654

[48] Kaiser R Leunig A Pekayvaz K Popp O Joppich M Polewka V . Self-sustaining IL-8 loops drive a prothrombotic neutrophil phenotype in severe COVID-19. JCI Insight. (2021) 6:e150862. doi: 10.1172/jci.insight.150862, PMID: 34403366 PMC8492337

[49] Del Valle DM Kim-Schulze S Huang HH Beckmann ND Nirenberg S Wang B . An inflammatory cytokine signature predicts COVID-19 severity and survival. Nat Med. (2020) 26:1636–43. doi: 10.1038/s41591-020-1051-9, PMID: 32839624 PMC7869028

[50] Heit B Tavener S Raharjo E Kubes P . An intracellular signaling hierarchy determines direction of migration in opposing chemotactic gradients. J Cell Biol. (2002) 159:91–102. doi: 10.1083/jcb.200202114, PMID: 12370241 PMC2173486

[51] Fuhler GM Knol GJ Drayer AL Vellenga E . Impaired interleukin-8-and GROα-induced phosphorylation of extracellular signal-regulated kinase result in decreased migration of neutrophils from patients with myelodysplasia. J leukocyte Biol. (2005) 77:257–66. doi: 10.1189/jlb.0504306, PMID: 15561756

[52] Trellakis S Bruderek K Dumitru CA Gholaman H Gu X Bankfalvi A . Polymorphonuclear granulocytes in human head and neck cancer: enhanced inflammatory activity, modulation by cancer cells and expansion in advanced disease. Int J cancer. (2011) 129:2183–93. doi: 10.1002/ijc.25892, PMID: 21190185

[53] Schimek V Strasser K Beer A Göber S Walterskirchen N Brostjan C . Tumour cell apoptosis modulates the colorectal cancer immune microenvironment via interleukin-8-dependent neutrophil recruitment. Cell Death Dis. (2022) 13:113. doi: 10.1038/s41419-022-04585-3, PMID: 35121727 PMC8816934

[54] Bolander J Moviglia Brandolina MT Poehling G Jochl O Parsons E Vaughan W . The synovial environment steers cartilage deterioration and regeneration. Sci Adv. (2023) 9:eade4645. doi: 10.1126/sciadv.ade4645, PMID: 37083524 PMC10121162

[55] Lopez-Ichikawa M Vu NK Nijagal A Rubinsky B Chang TT . Neutrophils are important for the development of pro-reparative macrophages after irreversible electroporation of the liver in mice. Sci Rep. (2021) 11:14986. doi: 10.1038/s41598-021-94016-8, PMID: 34294763 PMC8298444

[56] Phillipson M Kubes P . The healing power of neutrophils. Trends Immunol. (2019) 40:635–47. doi: 10.1016/j.it.2019.05.001, PMID: 31160208

[57] Devalaraja RM Nanney LB Qian Q Du J Yu Y Devalaraja MN . Delayed wound healing in CXCR2 knockout mice. J Invest Dermatol. (2000) 115:234–44. doi: 10.1046/j.1523-1747.2000.00034.x, PMID: 10951241 PMC2664868

[58] Nishio N Okawa Y Sakurai H Isobe K-i . Neutrophil depletion delays wound repair in aged mice. Age. (2008) 30:11–9. doi: 10.1007/s11357-007-9043-y, PMID: 19424869 PMC2276589

[59] Grenier A Chollet-Martin S Crestani B Delarche C El Benna J Boutten A . Presence of a mobilizable intracellular pool of hepatocyte growth factor in human polymorphonuclear neutrophils. Blood J Am Soc Hematology. (2002) 99:2997–3004. doi: 10.1182/blood.V99.8.2997, PMID: 11929792

[60] Deng Y Zhao Z Sheldon M Zhao Y Teng H Martinez C . LIFR recruits HGF-producing neutrophils to promote liver injury repair and regeneration. bioRxiv. (2023) 2023:2003. doi: 10.1101/2023.03.18.533289, PMID: 36993315 PMC10055204

[61] Jaffré S Dehoux M Paugam C Grenier A Chollet-Martin S Stern JB . Hepatocyte growth factor is produced by blood and alveolar neutrophils in acute respiratory failure. Am J Physiology-Lung Cell Mol Physiol. (2002) 282:L310–5. doi: 10.1152/ajplung.00121.2001, PMID: 11792636

[62] Brandel V Schimek V Göber S Hammond T Brunnthaler L Schrottmaier WC . Hepatectomy-induced apoptotic extracellular vesicles stimulate neutrophils to secrete regenerative growth factors. J hepatol. (2022) 77:1619–30. doi: 10.1016/j.jhep.2022.07.027, PMID: 35985549

[63] Gaudry M Brégerie O Vr A El Benna J Pocidalo M-A Hakim J . Intracellular pool of vascular endothelial growth factor in human neutrophils. Blood J Am Soc Hematology. (1997) 90:4153–61. doi: 10.1182/blood.V90.10.4153, PMID: 9354686

[64] Braile M Cristinziano L Marcella S Varricchi G Marone G Modestino L . LPS-mediated neutrophil VEGF-A release is modulated by cannabinoid receptor activation. J Leucocyte Biol. (2021) 109:621–31. doi: 10.1002/JLB.3A0520-187R, PMID: 32573828

[65] Gong Y Koh D-R . Neutrophils promote inflammatory angiogenesis via release of preformed VEGF in an *in vivo* corneal model. Cell Tissue Res. (2010) 339:437–48. doi: 10.1007/s00441-009-0908-5, PMID: 20012648

[66] Ohki Y Heissig B Sato Y Akiyama H Zhu Z Hicklin DJ . Granulocyte colony-stimulating factor promotes neovascularization by releasing vascular endothelial growth factor from neutrophils. FASEB J. (2005) 19:2005–7. doi: 10.1096/fj.04-3496fje, PMID: 16223785

[67] Christoffersson G Vågesjö E Vandooren J Lidén M Massena S Reinert RB . VEGF-A recruits a proangiogenic MMP-9–delivering neutrophil subset that induces angiogenesis in transplanted hypoxic tissue. Blood J Am Soc Hematology. (2012) 120:4653–62. doi: 10.1182/blood-2012-04-421040, PMID: 22966168 PMC3512240

[68] Loh JT Lam K-P . Neutrophils in the pathogenesis of rheumatic diseases. Rheumatol Immunol Res. (2022) 3:120–7. doi: 10.2478/rir-2022-0020, PMID: 36788971 PMC9895873

[69] Sato J Takahashi I Umeda T Matsuzaka M Danjyo K Tsuya R . Effect of alcohol drinking and cigarette smoking on neutrophil functions in adults. Luminescence. (2011) 26:557–64. doi: 10.1002/bio.1270, PMID: 21433278

[70] Saito Y Takahashi I Iwane K Okubo N Nishimura M Matsuzaka M . The influence of blood glucose on neutrophil function in individuals without diabetes. Luminescence. (2013) 28:569–73. doi: 10.1002/bio.2495, PMID: 23509074

[71] Beyrau M Bodkin JV Nourshargh S . Neutrophil heterogeneity in health and disease: a revitalized avenue in inflammation and immunity. Open Biol. (2012) 2:120134. doi: 10.1098/rsob.120134, PMID: 23226600 PMC3513838

[72] Blanter M Gouwy M Struyf S . Studying neutrophil function *in vitro*: cell models and environmental factors. J Inflammation Res. (2021) 2021:141–62. doi: 10.2147/JIR.S284941, PMID: 33505167 PMC7829132

[73] Brotfain E Hadad N Shapira Y Avinoah E Zlotnik A Raichel L . Neutrophil functions in morbidly obese subjects. Clin Exp Immunol. (2015) 181:156–63. doi: 10.1111/cei.12631, PMID: 25809538 PMC4469166

[74] Duarte M Kuchibhatla M Khandelwal S Arepally GM Lee GM . Heterogeneity in neutrophil responses to immune complexes. Blood Adv. (2019) 3:2778–89. doi: 10.1182/bloodadvances.2019000235, PMID: 31554616 PMC6784526

[75] Valerius NH Eff C Hansen NE Karle H Nerup J Søeberg B . Neutrophil and lymphocyte function in patients with diabetes mellitus. Acta Med Scandinavica. (1982) 211:463–7. doi: 10.1111/j.0954-6820.1982.tb01983.x, PMID: 6981286

[76] Neeli I Radic M . Opposition between PKC isoforms regulates histone deimination and neutrophil extracellular chromatin release. Front Immunol. (2013) 4:38. doi: 10.3389/fimmu.2013.00038, PMID: 23430963 PMC3576869

[77] Fetz AE Neeli I Rodriguez IA Radic MZ Bowlin GL . Electrospun template architecture and composition regulate neutrophil NETosis *in vitro* and *in vivo*. Tissue Eng Part A. (2017) 23:1054–63. doi: 10.1089/ten.TEA.2016.0452, PMID: 28068879

[78] Fetz AE King WE III Minden-Birkenmaier BA Bowlin GL . Methods for quantifying neutrophil extracellular traps on biomaterials. In: Biomedical engineering technologies, vol. 2. Midtown Manhattan, New York City, NY, USA: Springer (2022). p. 727–42. 10.1007/978-1-0716-1811-0_38 35094355

[79] Damascena HL Silveira WAA Castro MS Fontes W . Neutrophil activated by the famous and potent PMA (Phorbol myristate acetate). Cells. (2022) 11:2889. doi: 10.3390/cells11182889, PMID: 36139464 PMC9496763

[80] Minden-Birkenmaier BA Cherukuri K Smith RA Radic Z Bowlin GL . Manuka honey modulates the release profile of a dHL-60 neutrophil model under anti-inflammatory stimulation. J Tissue viability. (2020) 29:91–9. doi: 10.1016/j.jtv.2020.03.005, PMID: 32249090 PMC7239342

[81] Rincón E Rocha-Gregg BL Collins SR . A map of gene expression in neutrophil-like cell lines. BMC Genomics. (2018) 19:573. doi: 10.1186/s12864-018-4957-6, PMID: 30068296 PMC6090850

[82] Lawrence SM Corriden R Nizet V . The ontogeny of a neutrophil: mechanisms of granulopoiesis and homeostasis. Microbiol Mol Biol Rev. (2018) 82:1092–2172. doi: 10.1128/mmbr.00057-00017, PMID: 29436479 PMC5813886

[83] Bhakta SB Lundgren SM Sesti BN Flores BA Akdogan E Collins SR . Neutrophil-like cells derived from the HL-60 cell-line as a genetically-tractable model for neutrophil degranulation. PloS One. (2024) 19:e0297758. doi: 10.1371/journal.pone.0297758, PMID: 38324578 PMC10849234

[84] Bedner E Melamed MR Darzynkiewicz Z . Enzyme kinetic reactions and fluorochrome uptake rates measured in individual cells by laser scanning cytometry. Cytometry: J Int Soc Analytical Cytology. (1998) 33:1–9. doi: 10.1002/(SICI)1097-0320(19980901)33:1<1::AID-CYTO1>3.0.CO;2-P 9725553

[85] Kienle K Lämmermann T . Neutrophil swarming: an essential process of the neutrophil tissue response. Immunol Rev. (2016) 273:76–93. doi: 10.1111/imr.12458, PMID: 27558329

[86] Sanmamed MF Carranza-Rua O Alfaro C Onate C Martín-Algarra S Perez G . Serum interleukin-8 reflects tumor burden and treatment response across Malignancies of multiple tissue origins. Clin Cancer Res. (2014) 20:5697–707. doi: 10.1158/1078-0432.CCR-13-3203, PMID: 25224278

[87] Rajpathak SN Wang T Wassertheil-Smoller S Strickler HD Kaplan RC McGinn AP . Hepatocyte growth factor and the risk of ischemic stroke developing among postmenopausal women: results from the Women’s Health Initiative. Stroke. (2010) 41:857–62. doi: 10.1161/STROKEAHA.109.567719, PMID: 20203323 PMC3903044

[88] Palmer BR Paterson MA Frampton CM Pilbrow AP Skelton L Pemberton CJ . Vascular endothelial growth factor-A promoter polymorphisms, circulating VEGF-A and survival in acute coronary syndromes. PloS One. (2021) 16:e0254206. doi: 10.1371/journal.pone.0254206, PMID: 34260629 PMC8279389

[89] Johnston M McBride M Dahiya D Owusu-Apenten R Nigam PS . Antibacterial activity of Manuka honey and its components: An overview. AIMS Microbiol. (2018) 4:655. doi: 10.3934/microbiol.2018.4.655, PMID: 31294240 PMC6613335

[90] Alvarez-Suarez JM Gasparrini M Forbes-Hernández TY Mazzoni L Giampieri F . The composition and biological activity of honey: a focus on Manuka honey. Foods. (2014) 3:420–32. doi: 10.3390/foods3030420, PMID: 28234328 PMC5302252

[91] Yuslianti ER Sutarna TH Djohan FFS Hasna A . Formulation and antibacterial activity test of rambutan honey toothpaste against Streptococcus mutans. Med Sains: Jurnal Ilmiah Kefarmasian. (2024) 9:455–62. doi: 10.37874/ms.v9i2.1187

[92] Kwakman PH Te Velde AA de Boer L Vandenbroucke-Grauls CM Zaat SA . Two major medicinal honeys have different mechanisms of bactericidal activity. PloS One. (2011) 6:e17709. doi: 10.1371/journal.pone.0017709, PMID: 21394213 PMC3048876

[93] Almasaudi S . The antibacterial activities of honey. Saudi J Biol Sci. (2021) 28:2188–96. doi: 10.1016/j.sjbs.2020.10.017, PMID: 33911935 PMC8071826

[94] Li J D’Annibale-Tolhurst MA Adler KB Fang S Yin Q Birkenheuer AJ . A myristoylated alanine-rich C kinase substrate–related peptide suppresses cytokine mRNA and protein expression in LPS-activated canine neutrophils. Am J Respir Cell Mol Biol. (2013) 48:314–21. doi: 10.1165/rcmb.2012-0278OC, PMID: 23221047 PMC3604091

[95] Balakrishna S Song W Achanta S Doran SF Liu B Kaelberer MM . TRPV4 inhibition counteracts edema and inflammation and improves pulmonary function and oxygen saturation in chemically induced acute lung injury. Am J Physiology-Lung Cell Mol Physiol. (2014) 307:L158–72. doi: 10.1152/ajplung.00065.2014, PMID: 24838754 PMC4152165

[96] Schumacher C Clark-Lewis I Baggiolini M Moser B . High-and low-affinity binding of GRO alpha and neutrophil-activating peptide 2 to interleukin 8 receptors on human neutrophils. Proc Natl Acad Sci. (1992) 89:10542–6. doi: 10.1073/pnas.89.21.10542, PMID: 1438244 PMC50375

[97] Snyder AE Main EN Bowlin GL . Immunomodulation and mechanical characterization of manuka honey-incorporated near-field electrospun bioresorbable vascular grafts. Bioengineering. (2025) 12:1270. doi: 10.3390/bioengineering12111270, PMID: 41301226 PMC12649915

[98] Anderson JM . Biological responses to materials. Annu Rev materials Res. (2001) 31:81–110. doi: 10.1146/annurev.matsci.31.1.81, PMID: 41139587

[99] Fontayne A Dang PM-C Gougerot-Pocidalo M-A El Benna J . Phosphorylation of p47 p hox Sites by PKC α, βII, δ, and ζ: Effect on Binding to p22 p hox and on NADPH Oxidase Activation. Biochemistry. (2002) 41:7743–50. doi: 10.1021/bi011953s, PMID: 12056906

[100] Price MO McPhail LC Lambeth JD Han C-H Knaus UG Dinauer MC . Creation of a genetic system for analysis of the phagocyte respiratory burst: high-level reconstitution of the NADPH oxidase in a nonhematopoietic system. Blood J Am Soc Hematology. (2002) 99:2653–61. doi: 10.1182/blood.V99.8.2653, PMID: 11929750

[101] El-Benna J Dang PM-C Gougerot-Pocidalo M-A Marie J-C Braut-Boucher F . p47phox, the phagocyte NADPH oxidase/NOX2 organizer: structure, phosphorylation and implication in diseases. Exp Mol Med. (2009) 41:217–25. doi: 10.3858/emm.2009.41.4.058, PMID: 19372727 PMC2679237

[102] Tang ZH Peng J Ren Z Yang J Li TT Li TH . New role of PCSK9 in atherosclerotic inflammation promotion involving the TLR4/NF-κB pathway. Atherosclerosis. (2017) 262:113–22. doi: 10.1016/j.atherosclerosis.2017.04.023, PMID: 28535426

[103] Peng C Han J Ye X Zhang X . IL-33 treatment attenuates the systemic inflammation reaction in acinetobacter baumannii pneumonia by suppressing TLR4/NF-κB signaling. Inflammation. (2018) 41:870–7. doi: 10.1007/s10753-018-0741-7, PMID: 29508184

[104] Kong F Ye B Cao J Cai X Lin L Huang S . Curcumin represses NLRP3 inflammasome activation via TLR4/MyD88/NF-κB and P2X7R signaling in PMA-induced macrophages. Front Pharmacol. (2016) 7:369. doi: 10.3389/fphar.2016.00369, PMID: 27777559 PMC5056188

[105] Zou J Feng D Ling W-H Duan R-D . Lycopene suppresses proinflammatory response in lipopolysaccharide-stimulated macrophages by inhibiting ROS-induced trafficking of TLR4 to lipid raft-like domains. J Nutr Biochem. (2013) 24:1117–22. doi: 10.1016/j.jnutbio.2012.08.011, PMID: 23246157

[106] Kim C Dinauer MC . Impaired NADPH oxidase activity in Rac2-deficient murine neutrophils does not result from defective translocation of p47phox and p67phox and can be rescued by exogenous arachidonic acid. J leukocyte Biol. (2006) 79:223–34. doi: 10.1189/jlb.0705371, PMID: 16275890

[107] Thallas-Bonke V Thorpe SR Coughlan MT Fukami K Yap FYT Sourris KC . Inhibition of NADPH oxidase prevents advanced glycation end product–mediated damage in diabetic nephropathy through a protein kinase C-α–dependent pathway. Diabetes. (2008) 57:460–9. doi: 10.2337/db07-1119, PMID: 17959934

[108] Park J Shim MK Jin M Rhyu M-R Lee Y . Methyl syringate, a TRPA1 agonist represses hypoxia-induced cyclooxygenase-2 in lung cancer cells. Phytomedicine. (2016) 23:324–9. doi: 10.1016/j.phymed.2016.01.009, PMID: 26969386

